# Primary hyperparathyroidism in developing world: a systematic review on the changing clinical profile of the disease

**DOI:** 10.20945/2359-3997000000211

**Published:** 2020-03-18

**Authors:** Sanjay Kumar Yadav, Goonj Johri, Raouef Ahmed Bichoo, Chandan Kumar Jha, Ronald Kintu-Luwaga, Saroj Kanta Mishra

**Affiliations:** 1 Department of Breast and Endocrine Surgery Kalinga Institute of Medical Sciences Bhubaneswar Odisha India Department of Breast and Endocrine Surgery, Kalinga Institute of Medical Sciences, Bhubaneswar, Odisha, India; 2 Department of Endocrine Surgery Sanjay Gandhi Postgraduate Institute of Medical Sciences Lucknow India Department of Endocrine Surgery, Sanjay Gandhi Postgraduate Institute of Medical Sciences, Raebareli Road, Lucknow, India; 3 Department of General Surgery AIIMS Patna India Department of General Surgery, AIIMS, Patna, India; 4 Mulago National Referral Hospital Kampala Uganda Mulago National Referral Hospital, Kampala, Uganda

**Keywords:** Primary hyperparathyroidism, developing country, symptomatic PHPT, clinical spectrum of PHPT

## Abstract

While the developed world is focusing on laying guidelines for selecting out cases of Asymptomatic primary hyperparathyroidism (PHPT) for surgical intervention and promoting minimal access surgery, the developing world is observing a change in disease spectrum from advanced symptomatic to lesser degree of symptomatic disease and not many with associated Vitamin D deficiency. Few studies from the developing countries of the world have focused on the changing clinical spectrum of PHPT. Objective of this study is to review the changing profile of PHPT in developing world. A systematic literature search was done in December 2017 focussing on publications from the developing world. All studies pertaining to the epidemiology of PHPT published after 1^st^ January 2000 and published in English language were included for analysis. Most of the studies published from developing countries report a predominance of symptomatic disease (79.6% of all included patients) with musculoskeletal disease present in the majority of patients (52.9%). The combined mean serum total calcium (11.9 ± 1.4 mg/dL), serum PTH (668.6 ± 539 pg/mL), serum alkaline phoshpatase (619 ± 826.9 IU/L) and weight of excised parathyroid glands (4.4 ± 3.8 grams) are much higher than those reported from the western studies. Despite this, we found that there is a distinct trend towards a milder form of disease presentation and biochemical profile noticeable in more recent times. Although there is a striking difference in all aspects of PHPT disease epidemiology, clinical presentation and biochemical profile of developing and developed countries, there is a distinct trend towards a milder form of disease presentation and biochemical profile in more recent times.

## INTRODUCTION

Primary hyperparathyroidism (PHPT) emerged as a distinct clinical entity in 1920-1930. Since then the disease epidemiology and clinical-investigative protocols have witnessed a dramatic change. For the first 40 years after its recognition, the disease symptomatology was classically described as “stones, bones, moans, and groans”. However, the advent of a multichannel biochemical screening test in the early 1970s led to a clinical revolution in the diagnosis of PHPT with increased incidence by four to five- fold and appearance of the asymptomatic era of PHPT in developed countries (1).

Numerous publications between the years 1984 to 2010 from around the developing countries provided contrasting views on disease epidemiology and spectrum of clinical presentation (2-5). While the developed world focused on laying guidelines for filtering out cases of asymptomatic PHPT for surgical intervention and promoting minimal access surgery with the aid of newer diagnostic and localization tools, the developing world was observing a change in disease spectrum from advanced symptomatic to lesser degree of symptomatic disease and a reduction in incidence of associated Vitamin D deficiency. Some authors from developing countries have focused on the changing clinical spectrum of PHPT (6-10) and suggested that the trajectory of the change is similar to what was observed in developed countries a few decades earlier. We observed these dramatic changes in disease epidemiology and sought to review and address the question that whether the epidemiology of this disease continues to differ from the developed countries?

## MATERIALS AND METHODS

A systematic literature search on PubMed/Medline, Embase, Science Citation Index Expanded (SciSearch), Scopus and Cochrane Databases was done in December 2017 using keywords [“Primary hyperparathyroidism” AND “C”], where C is the name of a developing country according to WHO developing country stratification. The inclusion criteria were as follows: studies pertaining to the epidemiology of PHPT published after 1^st^ January 2000 and studies published in English language. Case reports, case series with less than ten subjects and studies on PHPT which didn’t report on the clinical/surgical/biochemical profile were excluded.

The study title, year of publication, sample size, patient demographics, disease characteristics, intervention (surgery vs medical management) and mean values of biochemical parameters [e.g. serum total calcium, parathormone (PTH), Vitamin D [25(OH)-Vitamin-D], creatinine, and phosphate were extracted from each study. Wherever required, the biochemical parameters were converted into thestandard units for the ease of comparison between studies.

## RESULTS

Developing countries lag behind developed countries in the number of publications. Majority of publications from developing countries are from India, China, Brazil, and Turkey. Publications from other developing countries are mostly case reports, small case series, and retrospective case series. These publications lack uniformity in the presentation, analysis, interpretation of data, and the specific information needed for a meta-analysis and appropriate statistical tests. The result of our search identified 115 publications from developing countries. After the exclusion of unrelated studies, case reports, and small case series, seventeen publications were eligible for review. All seventeen studies were observational in design and the details of the study are described in Table 1.

**Table 1:** Published studies from developing countries

S. No.	Author	Country	Period of study	No. of patients	Notable findings
1	Younes NA et al. (11)	Jordan	1990-2002	40	70% had skeletal disease
2	Ohe MN et al. (7)	Brazil	1985-2002	104	53% symptomatic
3	Malabu UH et al. (12)	Saudi Arabia	2000-2006	46	76% symptomatic
4	Prasarttong-Osoth P et al. (13)	Thailand	1997-2007	45	97.8% symptomatic
5	Zhao L et al. (14)	China	2000-2010	249	61.4% symtomaic
6	Paruk IM et al. (15)	South Africa	2003-2009	28	92.9% symptomatic
7	Eufrazino et al. (16)	Brazil	2007-2008	33	90% symtomatic but clasicalsymtoms only in 18.2%
8	Shah VN et al. (17)	India	1990-2010	202	99.1% symtomatic
9	Usta A et al. (18)	Turkey	1993-2013	190	72.0% symtomatic
10	Jha S et al. (19)	India	1994-2015	110	100% symtomatic
11	Bhadada et al. (20)	India	2005-2015	464	95% symtomatic
12	Ahsan T et al. (21)	Pakistan	2004-2014	25	96% symptomatic
13	Yadav SK et al. (10)	India	1990-2016	333	69% had musculoskeletal symtoms
14	Bahrami A et al. (22)	Iran	1985-2002	62	100% symptomatic
15	Bandeira F (23)	Brazil	Not reorted	90	57% symtomatic
16	Bandeira F (24)	Brazil	2000-2006	49	49% symtomatic
17	Bandeira FA (25)	Brazil	2000-2005	64	61% had skeletal or/and renal disease

### Demographics

Studies from South Africa (15) and Brazil (7) reported the mean age of presentation to be similar to that in the developed countries, although the vitamin D status of these patients was not reported. Studies from India, China, Pakistan, Iran and Turkey have reported median age of PHPT patients to be younger. Another interesting finding pertaining to the age of presentation was revealed in three Brazilian studies where the authors found that symptomatic patients presented at a much younger age than the asymptomatic ones (23-25). Seven studies, which overall included 1201 patients, reported on the vitamin D status of the patients (Table 2). The cumulative mean value of vitamin D for these patients is 22.0 ± 22.6 ng/mL, which falls into the category of ‘insufficient viamin D level’.

**Table 2:** Summary of biochemical parameters and gland weight in reviewed studies

S No	Country	Serum Total Calcium + SD (mg/dL)	Serum PTH + SD (pg/mL)	Serum alkaline phosphatase + SD (IU/L)	25-OH Vitamin-D + SD (ng/mL)	Mean weight of the gland + SD (Grams)
1	Jordan (11)	10.7 ± 1.2	438.2 ± 398	296.7 ± 245.9	NA	NA
2	Brazil (7)	12.0 ± 1.3	450.5 ± 376.6	N/A	N/A	NA
3	Saudi Arabia (12)	12.0 ± 0.4	440.0 ± 60.6	473 ± 146	14.3 ± 3.4	NA
4	Thailand (13)	13.1 (8.1–22)*	341 (107–2490)*	449 (35–3384)*	N/A	NA
5	China (14)	11.7 ± 1.4	402.1 (103.2–2700.6)*	112 (50–1419.9)*	13.0 (5.2–29.9)*	2.6 + 1.2
6	South Africa (15)	12.1 ± 1.6	340.9 ± 420.3	206.3 ± 340.2	N/A	2.6 ± 1.9
7	Brazil (16)	10.6 ± 1.3	182.5 ± 326.5	N/A	N/A	NA
8	India (17)	11.7 ± 1.5	740.2 ± 669.2	556.9 ± 551.4	25.0 ± 46.7	4.7 ± 1.6
9	Turkey (18)	11.9 (8.8–15.4)*	467 (102–2240)*	362 (64–685)*	N/A	NA
10	India (19)	NA	957.9 ± 245.4	NA	NA	NA
11	India (20)	11.9 ± 1.6	752.4 ± 735.2	653 ± 1180	22.9 ± 25.1	5.6 ± 6.5
12	Pakistan (21)	11.2 ± 1.6	879.5 ± 793.5	673.5 ± 729.6	16.3 ± 14.3	NA
13	India (10)	12.7 ± 1.6	715.9 ± 671.1	699.7 ± 725.7	20.7 ± 15.2	3.6
14	Iran (22)	11.2 (9.3-15.6)*	184 ± 41 (76–1300)	657 ± 116 (84–3150)	NA	NA
15	Brazil (23)	12.0 ± 0.5	714.1 ± 135.0	NA	NA	NA
16	Brazil (24)	11.3 ± 0.7	292.2 ± 179.4	NA	20.8 ± 8.9	NA
17	Brazil (25)	11.9 ± 0.2	289.7 ± 205.4	NA	21.4 + 2.4	3.7 + 2.5

*Mean values not reported in the study, median values with range provided.

### Clinical presentation

Symptomatic disease is still the most common form of presentation in developing countriesand 79.6% of all included patients were symptomatic (Tables 1 and 3). Musculoskeletal (52.9%) and renal disease (34.9%) were the most common reasons for presentation.It is hard to estimate the presence of neuropsychiatric disease in PHPT because they are rarely the presenting feature and may not be often recognized. This is manifested from our review as well, because only a few of the included studies reported the presence of neuropsychiatric disease in their patient cohort. Overall clinical presentation is summarised in Table 3.

**Table 3:** Overall clinical presentation in developing countries

Feature	Number of studies reporting the feature	Total number of patients for which data available	Total number of patient having the feature (%)
Symptomatic	17	2134	1699 (79.6)
Musculoskeletal disease	16	2030	1073 (52.9)
Renal disease	16	2030	709 (34.9)
Gastrointestinal disease	16	2030	211 (10.4)
Neuropsychiatric disease	16	2030	182 (08.9)

### Biochemical severity

Table 2 summarises the biochemical severity of PHPT in developing countries. As compared to developed nations, these patients presented with higher mean total serum calcium and PTH level. Mean total serum calcium level of 10.6 ± 0.6 mg/dL, mean PTH level of 67.5 (47.9-94.9) pg/mL and mean alkaline phosphatase (ALP) level of 75 (62-92) IU/L was reported from USA (26). In contrast, all studies from developing countries except two (15 & 31) have reported mean serum calcium above 11 mg/dL, mean PTH level above 340 pg/mL (except 31, 34 & 35; all Brazilian studies) and mean ALP level above 400 IU/L in most studies.

We calculated the combined mean values for serum total calcium, serum PTH and serum ALP level of all included studies (wherever reported) to get an overall picture of the disease severity. The combined mean values for serum total calcium (11.9 ± 1.4 mg/dL), serum PTH (668.6 ± 539 pg/mL) and serum ALP (619 ± 826.9 IU/L) are very different from what has been reported from the west. Mean weight of excised adenoma is also high in developing countries (4.4 ± 3.8 grams).

### Changing epidemiology

We could identify only five studies examining the changing disease pattern in developing countries, three from India, and one each from China and Brazil (Table 4). Shah and cols. analyzed data of 202 patients over two decades and divided into four different time periods, i.e. 1990 to 1994, 1995 to 1999, 2000 to 2004 and 2005 to 2010 (17). Although clinical presentations of PHPT (predominance of bone and stone symptoms) did not differ across different time periods and non-significant decrease in serum calcium levels at the time of diagnosis of PHPT was observed but a significant decline in the serum ALP level (P < 0.01) was found in the last decade, however, PTH level was higher in the last decade (P < 0.05).Jha and cols. reported that although clinical severity of the disease has decreased significantly, biochemical abnormalities of high PTH and ALP are still the same (19). Yadav and cols. grouped the PHPT patients into three subgroups based on the time span; 1990–1999, 2000–2009, 2010–2016, and clinical and biochemical parameters were compared (10). The clinical presentation in this study has evolved progressively with increase in older age group (35 vs 39 vs 43.85, p < 0.001), less patients with musculoskeletal symptoms (85.3 vs 76.8 vs 61%, p = 0.002) and less patients with severe bone disease (29.4 vs 10.7 vs 10.7%, p = 0.088). Biochemical parameters also showed a changing trend with a significant decrease in mean ALP level (1393 vs 965 vs 414.8 IU/L, p < 0.001) and serum PTH (837.52 vs 812.89 vs 635.74 pg/mL, p = 0.02). Ohe and cols. grouped 104 Brazilian patients into three groups, patients diagnosed from 1985-1989, 1990-1994, and 1995 to 2002 (7). Patients from the first group tended to be younger (mean ± SD, 43.0 ± 15 vs 55.1 ± 14.4 and 55.7 ± 17.3 years, respectively) and their mean serum calcium was significantly higher.Zhao and cols in their study of 249 Chinese PHPT patients reported that percentage of asymptomatic PHPT has increased from 21% in 2000–2006 to 42.4% to 52.5% in 2007–2010 (14).

**Table 4:** Studies from developing countries examining the change in pattern of PHPT

Study	Patient population	Findings
Shah VN et al. (17)	202 patients, divided into 4 time groups 1990-1994 (n = 6) 1995-1999 (n = 22) 2000-2004 (n = 43) 2005-2010 (n = 131)	Clinical presentation did not change over different time periods; non-significant decrease in serum calcium; significant decline in the serum alkaline phosphatase level in the last decade.
Jha S et al. (19)	110 patients, divided into 2 time groups: 1994-2007 (n = 51) 2007-2015 (n = 59)	Pathological fractures more common in earlier subgroup, bone pain was more common in later subgroup; median PTH values were similar in two time groups
Yadav SK et al. (10)	333 patients divided into 3 time groups: 1990–1999 (n = 34) 2000–2009 (n = 112) 2010–2016 (n = 187)	Progressive increase in age at presentation; decline in proportion of patients presenting with musculoskeletal disease; decline in mean serum PTH and serum alkaline phosphatase level
Ohe MN et al. (7)	104 patients divided into 3 subgroups 1985-1989 (n = 9) 1990-1994 (n = 30) 1995-2002 (n = 65)	Patients from earlier subgroup were significantly younger and had significantly higher serum calcium values
Zhao L et al. (14)	249 patients presenting between 2000-2010; Compared symtomatic and asymtomatic PHPT	The overall features of PHPT not changed but asymptomatic presentation increased to 50% of all cases after 2007; symptomatic patients had significantly higher serum calcium, PTH, alkaline phoshatase, and higher parathyroid gland weight.

## DISCUSSION

The presentation of PHPT has changed from symptomatic to an asymptomatic disease in developed countries (1). However, there is a striking difference in all aspects of PHPT disease epidemiology, clinical presentation and biochemical profile of patients from developing countries when compared to profile of patients from developed countries, but the clinical and biochemical severity is decreasing and is somewhat similar to observations on western patients reported during the1980s and 90s (27-29). One of the most interesting findings from this review pertains to the age of presentation. Patients of from developing Asian countries seem to present at a younger age, with a difference of about 15-20 years. The exact pathogenesis of this young age of presentation has not been clearly established, but the widespread prevalence of vitamin D deficiency in these countries, which has been linked with parathyroid adenoma growth, may be playing its part (27). Another theory is increased awareness in western countries (26) along with routine biochemical screeningbut only increased awareness can not explain the drastically opposite presentation of PHPT in developing countries. The cause of this type of presentation must be multifactorial like patient awareness, physician awareness, health infrastructure and nutrition.

Delay in diagnosis incurs high healthcare cost in the form of morbidities associated with PHPT. Education of general public and training of health care personnel can lead to early identification and management leading to patient benefit. As the awareness among general population and physicians is increasingthe diagnosis of PHPT is increasing in developing world. Increasing number of specialist endocrine surgeons is increasing across the globe but it is still very less in developing countries (30).

With the trajectory being similar to west in the terms of clinical and biochemical profile with a temporal lag of one and half to two decades, it can be expected that in another two decades, a steady state would be achieved, as in western patients.
